# Prevalence of depressive symptoms in patients with rheumatoid arthritis at a regional hospital in KwaZulu-Natal, South Africa

**DOI:** 10.4102/sajpsychiatry.v28i0.1702

**Published:** 2022-02-22

**Authors:** Mfundo Mabusela, Andrew Tomita, Saeeda Paruk, Farhanah Paruk

**Affiliations:** 1 Department of Internal Medicine, Faculty of Medicine, University of KwaZulu-Natal, Durban, South Africa; 2 Centre for Rural Health, School of Nursing and Public Health, University of KwaZulu-Natal, Durban, South Africa; 3 KwaZulu-Natal Research Innovation and Sequencing Platform (KRISP), College of Health Sciences, University of KwaZulu-Natal, Durban, South Africa; 4 Department of Psychiatry, Faculty of Medicine, University of KwaZulu-Natal, Durban, South Africa; 5 Department of Rheumatology, Faculty of Medicine, University of KwaZulu-Natal, Durban, South Africa

**Keywords:** rheumatoid arthritis, disease activity, depression, functional impairment, food insecurity

## Abstract

**Background:**

Depression affects 14.8% – 38.8% of patients with rheumatoid arthritis (RA) in developed countries. The prevalence and risk factors for depression in patients with RA in sub-Saharan Africa is not well established.

**Aim:**

To determine the prevalence of depressive symptoms in patients with RA.

**Setting:**

Public sector regional hospital in South Africa.

**Methods:**

A cross-sectional descriptive study was undertaken with 110 adult RA patients. A structured socio-demographic and clinical questionnaire, the modified health assessment questionnaire (mHAQ), the simplified disease activity index (SDAI) for RA, the patient health questionnaire (PHQ-9), and the Household Food Insecurity Access scale (HFIAS) for nutritional status, were used. Correlates of depressive symptomatology in participants with RA were identified using *t*-tests and regression analyses.

**Results:**

Most of the participants were women (90.9%), 67% had moderate to severe RA disease on the SDAI score, 92.7% reported functional disability (HAQ score of ≥ 1), and 87.2% reported mild to severe depressive symptoms. Unemployment (*p* < 0.01), severe food insecurity (*p* < 0.01) and functional disability (*p* = 0.02), were significantly associated with the depressive symptoms, but not with disease activity (*p* = 0.8) or inflammatory markers (*p* = 0.63). Unemployment (adjusted β = −5.07, *p* < 0.01) and severe food insecurity (adjusted β = −4.47, *p* < 0.01) were significantly associated with depressive symptoms, based on the adjusted regression model.

**Conclusion:**

As RA effects functional status, with the impact of the resulting unemployment and food insecurity being associated with depression, affected people should be screened for depression and managed using a multidisciplinary approach, especially considering the role of social determinants in RA patients with depression.

## Introduction

Untreated rheumatoid arthritis (RA) is associated with increased morbidity, mortality and poorer quality of life.^1,2^ Studies show that more than a third of formerly working persons are no longer able to perform work after 5 years of being diagnosed with RA, with this proportion increasing to more than 50% after 10 years.^3^ Affected persons not only have to find alternative sources of income, if possible, but are also often not able to perform their routine daily tasks and activities, which can result in inactivity,^4^ dependence on others and depression.

Depression is more common in persons with RA than in the general population, being estimated to affect 14.8% – 38.8% of those suffering from RA.^5^ Depressed persons with RA have poorer long-term outcomes, including pain, a higher number of comorbidities^6^ and increased mortality rates.^7^ The pathogenesis of depression in RA is not fully understood, however, it is postulated that immune dysregulation secondary to depression may play a central role in triggering the condition.^8^ The reticulo-limbic structures in the brain, which regulate pain and emotions, may also contribute to ongoing inflammation.^9^

Comorbid depression in RA is associated with a worsening of disease activity and severity,^10^ and has a significant impact on patient quality of life.^11^ Recent studies have shown a positive correlation between pain and depressive symptoms in RA.^12,13^ The condition impacts negatively on depression, and affected persons may experience perseverative thoughts, which is the process of uncontrolled repetitive negative thinking that results from the stress of their disease activity.^14^

There are limited studies on the prevalence and risk factors for depression amongst people with RA in Africa. Apart from high RA disease activity, which was identified in a Pakistani study,^15^ a small Tunisian study found that the main predictors associated with depression in RA patients were: female gender, absence of professional activity or social support, poor quality of life, and underlying structural joint damage.^16^ An Egyptian study showed that RA patients had significantly higher depression scores, were more likely to be clinically depressed compared to osteoarthritis patients, and were identified as being single and having a higher functional disability, when assessed by the health assessment questionnaire (HAQ), as significant predictors of clinical depression.^17^ A more recent observational cross-sectional study from Egypt involving 200 RA patients found that 42% had moderate to severe depression on the patient health questionnaire (PHQ-9) score, which correlated with age, disease duration, joint deformities, and inflammatory markers: C-reactive protein (CRP) and erythrocyte sedimentation rate (ESR).^18^

Solomon et al., in a study in Gauteng province, South Africa (SA), showed that in low-to-middle income countries (LMIC), such as SA, RA patients experienced substantially more depressive symptoms than those from developed countries, independent of age, sex, ethnic origin and disease characteristics.^19^ The potential impact of social circumstances, such as food insecurity and unemployment, were not explored in the study, although the authors postulated that social factors play an important role.

Although the association between food insecurity and depressive symptoms has not been explored amongst RA patients, a study from KwaZulu-Natal (KZN) province found that major depression was significantly associated with household food insecurity in patients with multi-drug resistance tuberculosis.^20^ Food insufficiency is associated with increased psychiatric morbidity,^21^ with a longitudinal study in the United States of America (US) reporting a simultaneous causal relationship between food insecurity and depression in a sample of rural, low-income women.^22^ Developing countries generally have higher rates of food insecurity because of poor socio-economic status. In addition, food insecure individuals tend to have inadequate management of chronic disease as a result of limited access to healthcare and or low health literacy or education attainment, even when controlled for education and income.^23,24^

Although many factors contribute to depression, unemployment has been consistently associated with higher rates in adults.^25,26^ A Lithuanian cross-sectional study found that long-term unemployment was associated with a higher likelihood of a depressed mood over a 12-month period.^27^ A study conducted in the US amongst young healthy adults aged 18 to 25 years, showed higher rates of depression in those unemployed (23%) than in those employed (12%).^28^

There are limited studies from developing countries about the prevalence and association of depressive symptoms in people living with RA. There are also few studies about the effects of social factors, such as unemployment, on depression in SA. This study will assist in improving the understanding of the association between depressive symptoms and RA, as well as predisposing clinical and social risk factors.

## Method

A cross-sectional, descriptive study of outpatients with RA attending a specialist arthritis clinic at King Edward VIII Hospital, a regional hospital in Durban, KZN province, was conducted from February to October 2019. The hospital services the urban and peri-urban areas of the eThekwini Municipality and offers specialist rheumatology and psychiatric services, amongst others. Patients seen are mainly black people and Indian (the second largest group), this demographic profile being reflected in our study cohort. According to the Census South Africa 2011, KZN had a population of 10.2 million people which comprises Africans (86.7%) followed by Indians/Asians (7.4%)^29^. Adult patients who were 18 years and older, diagnosed with RA, as per the American College of Rheumatology (ACR) criteria of 1987^30^ or 2010 ACR/European League Against Rheumatism (EULAR) criteria,^31^ on treatment for RA for at least 6 months, able and willing to consent for the study, and able to communicate in English or in isiZulu were randomly enrolled. Three patients refused to participate because of time constraint. Patients who were less than 18 years old, not willing to participate or unable to give informed consent, who had overlap syndromes and/or other auto-immune diseases and/or other inflammatory arthritis, were excluded.

### Measures

A structured questionnaire was used to collect demographic details (age, gender, ethnicity, marital status, educational level, and household monthly income) and clinical data, which was verified from the clinical records. Weight, height, smoking, human immunodeficiency virus (HIV) status, comorbid diseases, RA disease duration, serology, extra-articular manifestations, x-ray changes, RA disease activity scores and inflammatory markers were extracted from the clinical file. The inflammatory markers recorded were measured on the day of clinic visit. Body mass index (BMI) was classified according to the World Health Organization (WHO) classification.^32^ All self-reported questionnaires were available in English and isiZulu, with the interviews being conducted by the primary investigator (PI) who is bilingual, with the aid of a trained research assistant.

The modified HAQ (mHAQ), which is a patient reported tool, was used to measure both health status and health-related quality of life.^33^ The mHAQ tool is regarded as the gold standard for measuring physical disability in RA patients.^34^ It is a 20-item questionnaire that covers most activities of daily living (ADL) in eight domains, with score ranges from 0, indicating no disability, to a maximum of three, indicating dependence on others for all ADL. The mHAQ score is well validated for use in clinical care and correlates well with disease activity,^33^ having been used in other South African RA studies.^35,36^

Rheumatoid arthritis disease activity was measured using the simplified disease activity index (SDAI), which is a good predictor of joint damage and physical disability and widely used as an outcome measure.^37^ The score is a numerical summation of five outcome parameters: tender and swollen joint count (based on a 28-joint assessment), patient and physician global assessment of disease activity [visual analogue scale (VAS) 0–10 cm] and level of CRP (mg/dL, normal < 1 mg/dL). A SDAI score of < 3.3 indicates remission, 3.4–11.0 low disease activity, 11.1–26.0 moderate disease activity, and ≥ 26.1 high disease activity, and correlates RA disease activity with progression of joint damage.^38^ It has been validated by the South African Rheumatism and Arthritis Association, for scoring disease activity in RA,^39^ and has been used previously in the country.^40^

Depressive symptoms were assessed using the PHQ-9 tool, which has been validated for use in SA to identify comorbid depression in patients with chronic conditions.^41,42^ Depression was graded according to the PHQ-9 scores as follows: < 5 no depression; 5–10 mild depression; 11–14 moderate depression and >15 severe depression.

All participants identified with symptomatic depression were referred to the psychiatric unit for further evaluation.

The Household Food Insecurity Access scale (HFIAS) version 3 was used to measure food insecurity.^43^ The tool consists of a culturally invariant set of nine questions covering three related domains related to food insecurity and has been a widely used in KZN.^44,45,46^

The CRP within the last month, serology [rheumatoid factor (RF) and anti-citrullinated protein antibody (ACPA)] and HIV results were extracted from the patient records.

### Statistical analysis

Three analyses were conducted for this investigation, the first being the socio-demographic and clinical characteristics, which were summarised using proportions (%) for categorical and mean (and standard deviation–SD) for continuous variables. Second, we compared depressive symptomatology level by socio-demographic and clinical characteristics using a *t*-test. Third, we identified socio-demographic and clinical covariates of depressive symptomatology based on regression models. Given the small sample size, covariates found to be significant from the second analyses were fitted to the regression model, with all statistical analyses being performed using STATA 16.

### Ethical considerations

Ethical approval to conduct the study was obtained from the Biomedical Research Ethics Committee of the University of KwaZulu-Natal, (BE622/18), the hospital and the provincial Department of Health. The study was conducted according to the ethical guidelines and principles of the International Declaration of Helsinki and Guidelines for Good Clinical Practice in the Conduct of Clinical Trials with Human Participants in South Africa.

## Results

### Socio-demographic and clinical profile

Of the 113 patients with RA who were approached to participate 110 were enrolled, with a mean age of 56.6 years (SD ± 11.9 years), their socio-demographic profile and clinical characteristics are indicated in Table 1. The majority of the participants were female (*n* = 100, 90.9%), unemployed (*n* = 96, 87.3%) and reported severe food insecurity (*n* = 63, 57.3%).

**TABLE 1:** Socio-demographic and clinical characteristics of 110 participants with rheumatoid arthritis.

Characteristics	Total	
	*n*	%
**Age category (years):**		
25–45	15	13.6
46–59	46	41.8
60+	49	44.5
**Gender:**		
Female	100	90.9
**Ethnicity:**		
Black people	61	55.5
Indian	36	32.5
White people	8	7.3
Mixed race	5	4.5
**BMI categories (kg/m^2^):**		
Normal	29	26.4
Overweight	37	33.6
Obese	44	40
**Marital status:**		
Single/divorced/widowed	40	36.4
**Employment:**		
Yes	14	12.7
**Current smoking:**		
Yes	15	13.6
**Food insecurity:**		
None to moderate	47	42.7
Severe food insecurity	63	57.3
**Comorbidities:**		
Yes	59	53.6
**HIV infection:**		
Positive	8	7.3

BMI, body mass index; HIV, human immunodeficiency virus.

The disease activity, as per the SDAI score, functional status (HAQ) and serology (RF and ACPA), are shown in Table 2. The majority of participants (*n* = 74, 67%) had moderate to severe disease, according to their SDAI scores, and a total of 102 participants (92.7%) also reported severe functional disability, with a HAQ score of 1 or greater. An elevated CRP was found in 46 (41.8%) participants. Extra-articular manifestations were present in 24 (21.8%) participants and consisted of: anaemia (*n* = 15, 13.6%), osteoporosis (*n* = 5, 4.5%), interstitial lung disease (*n* = 2, 1.8%), Sjogren’s syndrome (*n* = 1, 0.9%) and uveitis (*n* = 1, 0.9%). Erosive disease on radiographs was seen in 49 (44.6%) patients, and all participants were on disease modifying anti-rheumatic drugs (DMARDS), and 59 (53.6%) were on two or more DMARDS.

**TABLE 2:** Rheumatoid arthritis disease activity profile in rheumatoid arthritis participants.

Characteristics	Total	
	*N*	%
**Simplified disease activity score (SDAI):**		
Low disease activity (≤11)	36	32.7
Moderate (12–26)	49	44.6
Severe (≥ 27)	25	22.7
**Functional status (HAQ):**		
Normal functioning (< 1)	8	7.3
Impaired functioning (≥ 1.1)	102	92.7
**Depression (PHQ-9 score):**		
Less than mild (≤ 4.9)	14	12.7
Mild (5 – 10.9)	21	19.1
Moderate (11 – 14.9)	28	25.5
Severe symptomatology (≥ 1 5)	47	42.7
**Extra articular features:**		
Yes	24	21.8
**Rheumatoid Factor:**		
Positive	87	79.1
**ACPA:**		
Positive	86	78.2
**C-reactive protein:**		
Normal (≤ 10.9)	64	58.2
Elevated (≥ 11)	46	41.8
**X-ray erosive disease:**		
Present	49	44.6
**DMARDS:**		
One	54	47.3
Two	46	41.8
Three	10	9.1

HAQ, health assessment questionnaire; ACPA, anti citrullinated peptide antibody;

DMARDS, disease modifying anti-rheumatic drugs.

### Depressive symptoms and socio-demographic and clinical features

Overall, 96 (87.2%) participants reported mild to severe depressive symptoms (score of 5 or more on PHQ 9). Tables 3 and 4 summarise the level of depressive symptoms based on the socio-demographic (Table 3) and clinical (Table 4) characteristics. The bivariate analysis based on the *t*-test shows that employment (*p* < 0.01), severe food insecurity (*p* < 0.01) and functional disability (HAQ score of ≥ 1), (p 0.02) were significantly associated with depressive symptom scores on the PHQ-9. There was no association between depressive symptom score and disease activity (*p* = 0.8) or CRP (*p* = 0.63). Unemployment (adjusted β = −5.07, *p* < 0.01) and severe food insecurity (adjusted β = −4.47, *p* < 0.01) were the only significant covariates associated with the depressive symptom scores, based on the adjusted regression model (model 1). When model 1 was controlled for basic socio-demographic variables, such as sex and gender, the significance of the finding did not change (model 2).

**TABLE 3:** Association of depression symptomatology by socio-demographic data in rheumatoid arthritis participants.

Socio-demographics	Depression symptomatology		Statistical result		
	Mean	SD	*t*	*df*	*p*
**Age category (years):**					
25–45	11.3	5.2	*F* = 0.48	2, 107	0.62
46–59	13.1	6.2	-	-	-
60+	12.5	6.5	-	-	-
**Gender:**					
Male	12.2	3.5	−0.22	108	0.82
Female	12.7	6.4	-	-	-
**Ethnicity:**					
Black	13.5	5.9	*F* = 1.26	2, 107	0.29
Indian	11.6	6.4	-	-	-
Mixed race/White	11.5	7.0	-	-	-
**Marital Status:**					
Single/divorced/widowed	12.2	6.0	−0.5	108	0.62
Married	12.8	6.4	-	-	-
**Employed:**					
No	13.4	5.9	3.79	108	<0.01
Yes	7.1	5.3	-	-	-
**Social Support:**					
No	13.6	6.7	1.41	108	0.16
Yes	11.9	5.9	-	-	-
**Food Insecurity:**					
None to moderate	9.4	6.3	−5.17	108	<0.01
Severe food insecurity	15	5.0	-	-	-

**TABLE 4:** Association of depression symptomatology by clinical characteristics.

Clinical characteristics	Depression symptomatology		Statistics		
	Mean	SD	*t*	*df*	*p*
**Body mass categories (kg/m^2^):**					
Normal	13	6.1	*F* = 0.08	2, 107	0.92
Overweight	12.4	6.3	-	-	-
Obese	12.6	6.4	-	-	-
**Currently smoking:**					
No	12.8	6.2	0.95	108	0.34
Yes	11.2	6.6	-	-	-
**HIV status:**					
Negative	12.4	6.3	−1.49	108	0.14
Positive	15.8	3.9	-	-	-
**Comorbidities:**					
No	12.1	5.6	−0.85	108	0.4
Yes	13.1	6.7	-	-	-
**Extra articular features:**					
No	12.6	6.1	0.07	108	0.95
Yes	12.5	6.8	-	-	-
**Functional Status (HAQ):**					
Normal function (< 1)	7.8	5.4	−2.34	108	0.02
Low functional (≥ 1)	13	6.1	-	-	-
**Disease activity (SDAI):**					
None to low (≤ 11)	10.7	6.4	−0.25	108	0.8
Moderate (12–26)	13.4	6.2	-	-	-
Severe (≥ 27)	13.9	5.6	-	-	-
**C-Reactive Protein Level (CRP):**						
Normal (≤ 10)	12.9	5.98	0.48	108	0.63
Severe (≥ 11)	12.3	6.58	-	-	-
**Rheumatoid factor:**					
Negative	12.6	6.18	−0.05	108	0.96
Positive	12.6	6.26	-	-	-
**Anti-citrullinated peptide antibody:**						
Negative	12.3	6.15	−0.25	108	0.8
Positive	12.7	6.26	-	-	-
**X-ray erosive disease:**					
No abnormality	12.2	6.28	−0.82	108	0.41
Abnormal	13.2	6.15	-	-	-
**Medication:**					
None or one	12.9	5.72	0.44	108	0.66
Two or more	12.4	6.65	-	-	-
Obese	12.6	6.39	-	-	-

HIV, human immunodeficiency virus.

## Discussion

This is the first study to describe the prevalence and association of depressive symptoms and food insecurity in patients with established RA. The main study findings are that there was a high prevalence of depressive symptoms in patients with RA, and that these were associated with unemployment (*p* < 0.01), severe food insecurity (*p* < 0.01), and impaired functional level [HAQ score 1 or more] (*p* < 0.02). However, the logistic regression showed that only unemployment and severe food insecurity were associated with depressive symptoms, and that there was no association with any clinical variables of RA, including CRP levels.

The prevalence of depression in this study was 87.3%, which was significantly above the 12-month prevalence of 4.5% reported for the general population in the South African Stress and Health Study (SASH),^47^ and greater than the prevalence of 38% reported in a meta-analysis reviewing 72 studies that included 13 189 patients living with RA.^5^ The rate was also higher than the 18% and 30% prevalence reported for minor depression or dysthymic disorder, respectively, in RA patients in a Chinese systemic review.^48^ The prevalence rate also exceeded studies from Tunisia and Egypt, which were 45%^17^ and 42%,^19^ respectively. The use of different methods or tools to estimate depression prevalence may account for the differences observed.

An earlier study by Solomon et al. in South African RA patients also found a higher burden of depression in the public than private sector using the Arthritis Impact Depression Scale (AIMS) screening tool.^20^ The high prevalence rate reported in this study was may be because of the use of a screening rather than a diagnostic tool, overlap between depressive and RA somatic symptoms, or subjective over reporting by participants. However, the results suggest the urgent need for inclusion of mental health screening services for this population.

Solomon’s study also suggested that depression was more common in RA patients in LMIC than previously thought, and postulated that social circumstances and comorbidities may play an important role in predicting depression in this cohort.^21^ The finding of depressive symptoms being associated with unemployment in this study is consistent with those reported elsewhere^28,46,49^ in people living with RA. The majority of the patients who participated in our study had low socio-economic circumstances and education levels.^50^ Unemployment in patients with RA is significantly affected by the stage and duration of RA, and increases with disease duration;^3^ our findings suggests that this may drive their depressive symptom development.

In this study, 57.3% of patients also reported severe food insecurity, which was associated with depressive symptoms, the two being strongly associated in low-income areas.^51,52^ This finding highlights that social factors may increase depression risk,^53^ which then impacts on treatment adherence and quality of life, as seen in other chronic disorders, such as HIV infection.^54^

This study, similar to other cross-sectional and retrospective studies conducted previously in Africa, including in SA,^20,36^ also supported the finding that there was a high level of RA disease activity and functional disability amongst people living with the condition. Having difficulty performing functional tasks was associated with a higher occurrence of depressive symptoms amongst Chinese middle aged and older adults^55^ and in a Pakistani study.^56^ In this study, the majority of patients had functional impairment, with the bivariate analysis suggesting that their functional level was associated with depressive scores similar to other studies.^17,18^ However, it was not significant on logistic regression, indicating the need to further explore this in a larger study.

Although most of the patients had moderate to severe disease activity, based on their SDAI score, erosive disease and raised CRP, these were not associated with depressive symptoms. In contrast, in the Tunisian study, the main predictors for depression in RA patients were high RA disease activity, impaired quality of life and existence of structural change.^17^ Similarly, an Egyptian study also found significant positive correlations between hospital anxiety depression scale-depression subscale (HADS-D) and age, disease duration, HAQ score, visual analogous scale for pain, RA Disease Activity Score-28 (DAS 28) and CRP levels.^19^ The lack of association between depression and functional impairment, disease activity scores, inflammatory markers or radiological changes in this study may be because of social factors driving the condition in this LMIC setting, where patients receive specialist medical level care but are negatively affected by economic challenges. The findings suggest that adverse socio-economic factors may be relevant in depressive symptom risk, as indicated in the study by Solomon et al.^20^

## Limitations

This is a single centre urban hospital-based study, with a small sample size of outpatients treated in a public health sector arthritis clinic, thus limiting the generalisability of findings to other settings. This could result in a bias of the results towards variables affected by poor socio-economic circumstances, for example food insecurity.

## Conclusions

The high prevalence of depressive symptoms amongst the patients with RA is concerning, and the association with unemployment and severe food insecurity suggests the need for regular depression screening, as well as the need to address social issues at chronic medical outpatient services. The study findings highlight the need to consider psycho-social and clinical factors that may influence health outcomes, and hence the need to develop more integrated services that includes a multidisciplinary management approach with medical, mental health and social work services. Larger, multicentre, longitudinal quantitative and qualitative studies are needed to better understand the factors associated with depressive symptoms in this clinical population.
